# Supplementary material for: The push-to-open mechanism of the tethered mechanosensitive ion channel NompC
Source: eLife. 2021 Jun 8;10:e58388. doi: 10.7554/eLife.58388 (PMC8186909; doi:10.7554/eLife.58388)
Supplement: Supplementary file 1. — (a) Molecular dynamics/steered molecular dynamics (MD/SMD) trajectories of system I. (b) MD/SMD trajectories of system II. (c) Ion permeation simulations of the partially opened NompC. (d) Stable hydrogen bonds and their occupancies in the MD/SMD trajectories. (e) Different domains/components used in the MD simulations. (f) The primers used for the alanine substitution. [file elife-58388-supp1.docx]

**SUPPLEMENTARY TABLES**

**Supplementary file 1a**. MD/SMD trajectories of system I.

| Trajectory (Label) | Pulling Group | Pulling Speed (nm/ns) | Force Constant  (kJ·mol^-1^nm^-2^) | Force Type | Simulation Time (ns) |
| --- | --- | --- | --- | --- | --- |
| Free I 0 (FI0) | / | 0 | 0 | / | 500 |
| Compress I 0 (CI0) | AR29 | 0.01 | 100 | pushing | 250 |
| Stretch I 0 (SI0) | AR29 | 0.01 | 100 | pulling | 250 |
| Free I 1-3  (FI1-3) | / | 0 | 0 | / | 500 × 3 |
| Compress I 1-3 (CI1-3) | AR29 | 0.005 | 50 | pushing | 500 × 3 |
| Stretch I 1-3  (SI1-3) | AR29 | 0.005 | 50 | pulling | 500 × 3 |
| D1236A | / | / | / | / | 500 |
| E1571A | / | / | / | / | 500 |
| W1115A | / | / | / | / | 500 |

**Supplementary file 1b**. MD/SMD trajectories of system II.

| Trajectory (Label) | Pulling Group | Force (pN) | Force Type | Simulation Time (ns) |
| --- | --- | --- | --- | --- |
| Free II 1 (FII1) | / | / | / | 40 |
| Compress II 1 (CII1) | AR1 | 5 | pushing | 40 |
| Stretch II 1 (SII1) | AR1 | 5 | pulling | 40 |
| Free II 2-6 (FII2-6) | / | / | / | 0.04 × 5 |
| Compress II 2-6 (CII2-6) | AR1 | 5 | pushing | 0.04 × 5 |
| Stretch II 2-6 (SII2-6) | AR1 | 5 | pulling | 0.04 × 5 |
| Free II 7 Chain A (FII7) | AR1 Chain A | / | / | 100 |
| Compress II 7 Chain A (CII7) | AR1 Chain A | 5 | pushing | 100 × 3 |
| Stretch II 7 Chain A (SII7) | AR1 Chain A | 5 | pulling | 100 × 3 |
| Free II 8-9 (FII8-9) | / | / | / | 100 × 2 |
| Stretch II 8-9 (SII8-9) | AR1 | 1 | pulling | 100 × 2 |
| Stretch II 10-11 (SII10-11) | AR1 | 2 | pulling | 100 × 2 |
| Stretch II 12-13 (SII12-13) | AR1 | 3 | pulling | 100 × 2 |
| Stretch II 14-15 (SII14-15) | AR1 | 4 | pulling | 100 × 2 |
| Stretch II 16-17 (SII16-17) | AR1 | 5 | pulling | 100 × 2 |
| Compress II 8-9 (CII8-9) | AR1 | 1 | pushing | 100 × 2 |
| Compress II 10-11 (CII10-11) | AR1 | 2 | pushing | 100 × 2 |
| Compress II 12-13 (CII12-13) | AR1 | 3 | pushing | 100 × 2 |
| Compress II 14-15 (CII14-15) | AR1 | 4 | pushing | 100 × 2 |
| Compress II 16-17 (CII16-17) | AR1 | 5 | pushing | 100 × 2 |

**Supplementary file 1c**. Ion permeation simulations of the partially opened NompC.

| Trajectory | Transmembrane Potential (mV) | Simulation Time (ns) |
| --- | --- | --- |
| Permeation I (PI1-3) | -300 | 200 × 3 |
| Permeation II (PII1-3) | 300 | 200 × 3 |

**Supplementary file 1d.** Stable hydrogen bonds and their occupancies in the MD/SMD trajectories.

| Residue 1 | Residue 2 | Free | Stretch | Compress |
| --- | --- | --- | --- | --- |
| D1236 (LH) | R1581 (TRP) | 98% | 97% | 98% |
| S1421 (4S5) | W1572 (TRP) | 60% | 81% | 81% |
| Q1253 (LH) | S1577 (TRP) | 56% | 44% | 73% |
| K1244 (LH) | E1571 (TRP) | 94% | 94% | 97% |
| W1115 (AR29) | D1142(LH) | 88% | 91% | 91% |
| R1127 (AR29) | E1163(LH) | 91% | 90% | 90% |

**Supplementary file 1e.** Different domains/components used in the MD simulations.

| Domain/Component of NompC | Residue Range |
| --- | --- |
| Ankyrin Repeats (ARs) | M124 - T1136 |
| Linker Helix (LH) | Y1137 - S1263 |
| Transmembrane domain (TM) | L1264 - W1602 |
| TRP domain (TRP) | Q1562 - H1584 |
| Ankyrin Repeat 1 (AR1) | M124 - N160 |
| Ankyrin Repeat 29 (AR29) | Y1109 - T1136 |
| 4S5 Linker (4S5) | L1420 - L1436 |

**Supplementary file 1f.** The primers used for the alanine substitution.

| Mutation | Sequence |
| --- | --- |
| D1236A-F | gcgaaacgtggagtttctcgccgttctcattgaaaatgagca |
| D1236A-R | cgagaaactccacgtttcgc |
| Q1253A-F | tgattgcccacacggtagttgcgcgatacttgcaagaactct |
| Q1253A-R | aactaccgtgtgggcaatca |
| W1572A-F | aggcccaatccgacatcgaggcgaaatttggcttgtccaagc |
| W1572A-R | ctcgatgtcggattgggcct |
| S1421A-F | tgcagatcctcgactttttgtcctgccaccacctattcg |
| S1421A-R | aggacaaaaagtcgaggatctgca |
| S1577A-F | tcgagtggaaatttggcttggccaagcttatacgcaatatgc |
| S1577A-R | caagccaaatttccactcga |
| K1244A-F | ttctcattgaaaatgagcaggcggaagtgattgcccacacg |
| K1244A-R | ctgctcattttcaatgagaac |
| E1571A-F | tcaggcccaatccgacatcgcgtggaaatttggcttgtcca |
| E1571A-R | gatgtcggattgggcctga |
| W1115A-F | actacggttgcgccgccattgcgttcgccgcctccgagg |
| W1115A-R | aatggcggcgcaaccgtagt |
| R1127A-F | agggacacaacgaggtcctggcgtatctgatgaacaaggagca |
| R1127A-R | caggacctcgttgtgtccct |
| D1142A-F | cacctacggcctgatggaggccaagcgattcgtgtacaac |
| D1142A-R | cctccatcaggccgtaggtg |
| E1163A-F | caacaacaagcccattcaggcgtttgtcctggtatcacca |
| E1163A-R | cctgaatgggcttgttgttg |
| R1581A-F | ttggcttgtccaagcttatagccaatatgcatcgcaccaca |
| R1581A-R | tataagcttggacaagccaaatttc |
